# Scaffolding depth cues and perceptual learning in VR to train stereovision: a proof of concept pilot study

**DOI:** 10.1038/s41598-021-89064-z

**Published:** 2021-05-12

**Authors:** Angelica Godinez, Santiago Martín-González, Oliver Ibarrondo, Dennis M. Levi

**Affiliations:** 1 School of Optometry, University of California, Berkeley, USA; 2 Construcción E Ingeniería de Fabricación, Universidad de Oviedo, Oviedo, Spain; 3 RS-Statistic, Arrasate-Mondragón, Spain

**Keywords:** Human behaviour, Perception

## Abstract

Stereopsis is a valuable feature of human visual perception, which may be impaired or absent in amblyopia and/or strabismus but can be improved through perceptual learning (PL) and videogames. The development of consumer virtual reality (VR) may provide a useful tool for improving stereovision. We report a proof of concept study, especially useful for strabismic patients and/or those with reduced or null stereoacuity. Our novel VR PL strategy is based on a principled approach which included aligning and balancing the perceptual input to the two eyes, dichoptic tasks, exposure to large disparities, scaffolding depth cues and perception for action. We recruited ten adults with normal vision and ten with binocular impairments. Participants played two novel PL games (DartBoard and Halloween) using a VR-HMD. Each game consisted of three depth cue scaffolding conditions, starting with non-binocular and binocular cues to depth and ending with only binocular disparity. All stereo-anomalous participants improved in the game and most (9/10) showed transfer to clinical and psychophysical stereoacuity tests (mean stereoacuity changed from 569 to 296 arc seconds, *P* < 0.0001). Stereo-normal participants also showed in-game improvement, which transferred to psychophysical tests (mean stereoacuity changed from 23 to a ceiling value of 20 arc seconds, *P* = 0.001). We conclude that a VR PL approach based on depth cue scaffolding may provide a useful method for improving stereoacuity, and the in-game performance metrics may provide useful insights into principles for effective treatment of stereo anomalies.

This study was registered as a clinical trial on 04/05/2010 with the identifier NCT01115283 at ClinicalTrials.gov.

## Introduction

Our rich perception of depth provides important information for navigation^1^ and action^2,3^. Depth perception is a complex process which requires the brain to integrate different visual cues^4^. Of those cues, many require only one eye (non-binocular cues) and include overlapping (interposition), perspective (conical projection), lighting-shading, chromatic attenuation, focus and motion parallax (created by the relative motion between an observer’s head and the perceived scene).

Stereopsis plays a key role in extracting depth information from natural scenes^5^, breaking camouflage^6^, and planning and executing everyday visuomotor tasks^1–3^. However, abnormal visual experience during the “sensitive period” of development^7,8^ may result in amblyopia and, as a result, in reduced or absent stereopsis^9^. Amblyopia, the leading cause of visual loss in children, is a neuro-developmental disorder arising from an imbalance between the ocular inputs to the visual pathway^10–12^. It is characterized as reduced visual acuity in an otherwise normal eye despite best optical correction^13^ and is typically secondary to misalignment of the visual axis (strabismus) and/or unequal refractive error (anisometropia).

Under everyday conditions, the loss of stereopsis is the most significant issue for individuals with amblyopia and strabismus, affecting their ability to reach and grasp^3,14^, navigate safely and rapidly^1^ and play certain sports^15^. Indeed, a recent analysis suggests that ≈ 7% of the population may be stereoblind^16^. Thus, for the overall well-being of people with amblyopia, stereopsis may be an important function to recover and/or strengthen. Perceptual Learning (PL), defined as “any relatively permanent and consistent change in the perception of a stimulus array following practice or experience with this array…”^17^, has demonstrated great potential in amblyopia treatment^18^. Although functionally suppressed when viewing binocularly^19,20^, binocular mechanisms seem to be intact in some people with amblyopia^21–23^, making stereo training a viable option. A number of different approaches have been evaluated for the recovery of stereopsis when compromised by amblyopia^24^. However, it is important to point out that stereopsis is more impacted in strabismic than in anisometropic amblyopia^24,25^ and recovery may require more active treatment^24^. Interestingly, patients with strabismic amblyopia benefit more from dichoptic training compared to monocular training and fare even better with direct training^24^. Furthermore, people with normal binocular vision can also benefit from training, improving their stereoacuity thresholds^26–28^. However, laboratory-based paradigms require participants to sit through many hours of monotonous psychophysical training^29,30^.

Considering that the main drawbacks of laboratory-based training paradigms are participant compliance, attention and motivation, several authors have proposed the use of specifically designed video games to treat amblyopia. Gamification, i.e. the use of game principles in non-game contexts, include the use of levels of increasing difficulty adapted to participant performance, rewards, a story line, and social context, among other aspects^31–33^. Several laboratory studies have reported benefits of using video games to treat amblyopia^34–39^, including direct stimulation of stereopsis^40^.

Recent commercialization of VR-HMDs has encouraged the design of therapies that incorporate gamification principles and builds upon successful laboratory-based techniques such as PL and dichoptic training. VR-HMDs provide the ability to present separate images to each eye, correct through software misalignment due to strabismus and adjust contrast or luminance independently for each eye until balanced binocular vision is achieved. VR-HMDs provide a wide visual field, facilitating vergence in users with strabismus, and large disparities that may help improve stereopsis^41,42^. Furthermore, VR-HMDs provide the ability to control depth cue content, with the exception of accommodation, which is the only cue without a commercial solution^43^. Depth cue content control facilitates design treatments based on a cue scaffolding strategy, assuring engagement in PL activities. This may be especially important for patients with poor to null stereopsis, who would become very frustrated by failing on a game with only binocular (disparity) cues. Indeed, Ding and Levi (2011) paired an informative monocular position cue with their disparity cue based on the hypothesis that patients with poor stereopsis have relied primarily on non-stereo depth cues, and with practice and feedback, patients could learn to increase reliance on the stereo information. Similarly, Vedamurthy et al. (2016), paired disparity cues with perspective cues while providing rich feedback and demonstrated that participants with poor or no stereopsis learned to upweight reliance on the stereo information.

Our aim was to develop and pilot two VR games that combine demanding stereovision tasks, dichoptic PL and depth cue scaffolding. The games were designed to incorporate nine principles: (1) alignment of images on corresponding areas in the two eyes, and (2) balancing the perceptual input to facilitate fusion; (3) combining non-binocular and binocular cues to depth as a ‘scaffold’ for depth judgements and systematically reducing the non-binocular cues; (4) exposure to large binocular disparities. Recent work has shown that viewing engaging and immersive 3D action videogames with large disparities improved stereoscopic vision in both amblyopic and neurotypical observers^41,42^. (5) A dichoptic anti-suppression task (6) requiring depth perception for action; (7) rich feedback, (8) ability to track in-game performance, including (9) trial-by-trial tracking. The latter requirement enabled us to compare the evolution of depth perception under different cue conditions.

## Results

### Changes in stereoacuity

**Figure 1:**
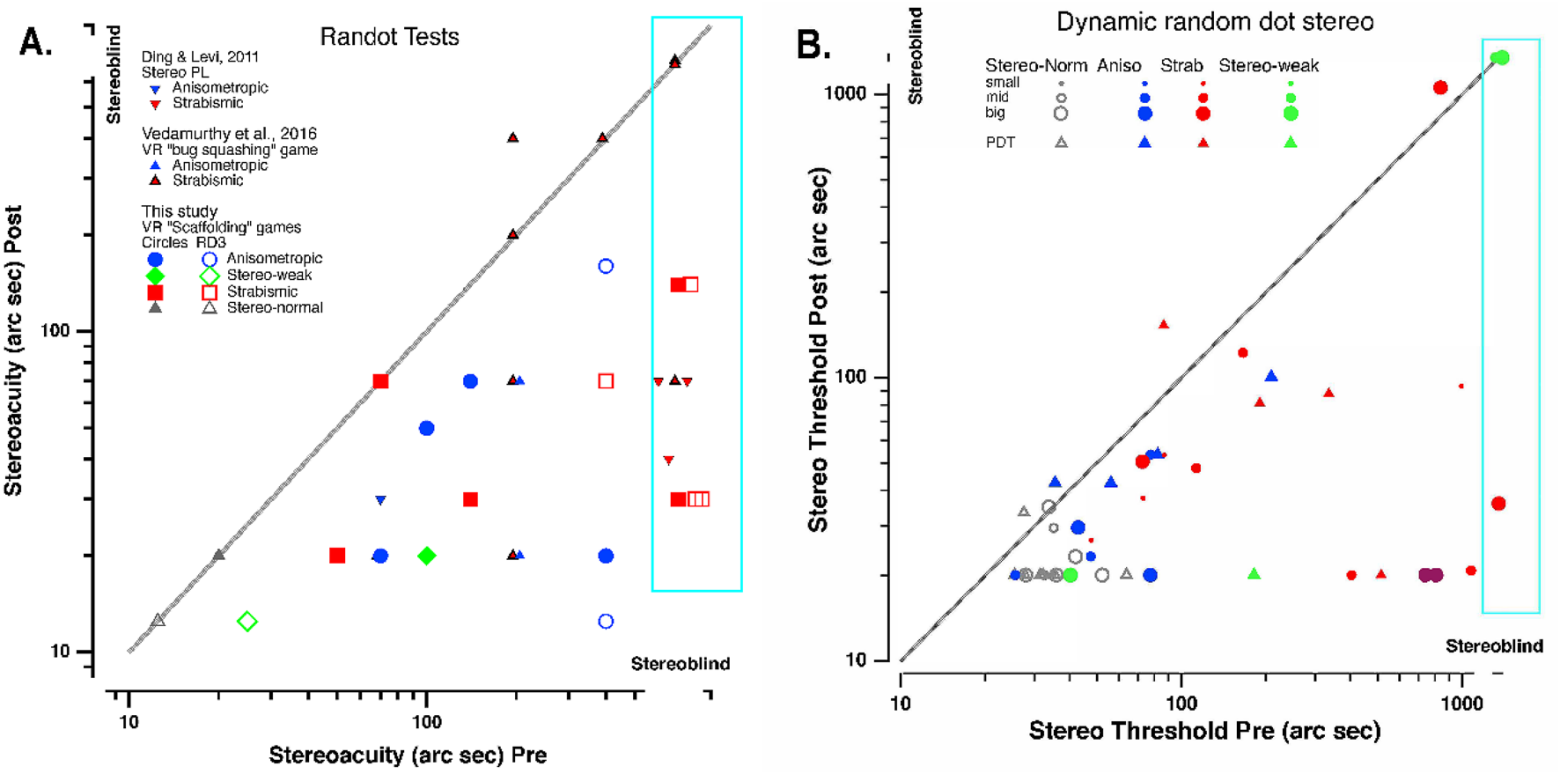
Stereoacuity transfer after training for clinical and psychophysical tests. (**A**) Improvement in clinical stereoacuity as a function of initial RCS (filled symbols) and RD3 (open symbols) threshold and comparison with Ding & Levi, 2011 (upside-down triangles) and Vedamurthy et al., 2016 (right-side up triangles). (**B**) Psychophysical stereoacuity improvement as a function of initial stereo threshold for PDT (triangle), DRS small (small circle), DRS medium (medium circle) and DRS big (big circle). In both figures, colors indicate binocular condition: anisometropia (blue), strabismus (red), stereo-weak (green), and normal stereo (grey). Data under the unity line indicate an improvement in stereoacuity.

The most important result of this study is the improvement of stereoacuity after training, particularly in the stereo-anomalous group (Fig. 1). Note that several participants were “stereoblind” (i.e., unable to identify the largest disparity presented) initially, but showed measurable stereoacuity after training.

We first analyzed the mean difference (before and after treatment) for each test between the stereo-normal and stereo-anomalous groups (Table 1). We found statistically significant differences between the stereo-normal and stereo-anomalous groups in both pre- and post-analysis for all tests, except for the Dynamic Random-dot Stereogram test (DRS) small pre- treatment (*P* = 0.052) and DRS big post- treatment (*P* = 0.100). When comparing pre- and post- results, although median results differ in the stereo-anomalous group clearly, significant differences occurred only in the Pure Disparity Test (PDT) for stereo-normal participants and in the Randot Circles Stereotest (RCS) test for stereo-anomalous participants.

**Table 1:** Pre and post median stereoacuity thresholds for each group and stereoacuity test and statistical mean comparison (*P*-value).

	Pre (median)		Post (median)		*P*-value Normal vs Anomalous		*P*-value Pre vs Post	
	Normal	Anomalous	Normal	Anomalous	Pre	Post	Normal	Anomalous
RCS	20.0"	100.0"	20.0"	30.0"	< 0.001*	0.006*	-	0.004*
RD3	12.5"	400.0"	12.5"	89.4"	< 0.001*	0.002*	-	0.164
PDT	29.5"	185.6"	20.1"	66.0"	< 0.001*	0.002*	0.017*	0.054
DRS small	20.1"	59.1"	20.1"	23.6"	0.052	0.006*	0.078	0.507
DRS medium	20.1"	93.6"	20.1"	22.1"	0.004*	0.045*	0.871	0.168
DRS big	23.7"	239.6"	20.1"	24.4"	0.003*	0.100	0.121	0.055

The clinical tests show a clear improvement in stereoacuity for stereo-anomalous participants (Fig. 1A), but not for stereo-normal since stereo-normal participants were at ceiling. The psychophysical tests, with neither monocular nor non-stereoscopic binocular cues, reveal significant improvements for both groups (Fig. 1B and Table 1), even though the differences between pre- and post-treatment show no statistical significance.

Adopting the criteria for stereoacuity improvement as an improvement of at least two levels on the clinical tests and a final stereoacuity threshold of 140 arc secs or better^24^, all participants in the stereo-anomalous group except for ASM1 improved in the RCS test, and all but ASM1, AS1, AS2 and AS4 improved in the Random Dot 3 Stereo Acuity Test with Lea Symbols (RD3).

Participant AMS1 failed to improve according to both clinical tests, although PDT and DRS small show an improvement. Participant AS1 failed to improve according to the RD3, but improved on all the other tests. Participant AS2 did not exhibit improvement with either the RD3 or the PDT (small regression) but showed improvements with all the other tests. Finally, participant AA3 appeared to regress in the PDT, but improved according to all other tests.

Seven of the ten participants in the stereo-normal group showed improvements in the PDT: N9 and N10 exhibited a regression and N1 was at ceiling. Similarly, seven of the ten participants showed improvements in DRS: N3 and N5 were at ceiling and N2 exhibited a slight regression in DRS big. Those improvements were not evident in the clinical tests because all stereo-normal participants were at ceiling before treatment.

Lastly, we analyzed whether the initial stereoacuity predicted the magnitude of improvement in stereoacuity following training (Table 2). Our analysis reveals strong and significant correlations between the initial psychophysical stereoacuity threshold and the amount of improvement (i.e., the Pre:Post stereoacuity ratio [PPR]) for the stereo-normal group, but failed with the clinical stereoacuity tests, as the participants were at ceiling. For the stereo-anomalous group the correlations are moderate and not statistically significant.

**Table 2:** Correlation coefficients and p-values using Pearson’s test between PPR and initial stereoacuity values for stereo-normal and stereo-anomalous groups.

Test	Stereo-normal		Stereo-anomalous	
	Correlation	*P*-value	Correlation	*P*-value
RCS	-	-	0.49	0.155
RD3	-	-	− 0.18	0.625
PDT	0.92	< 0.001*	0.63	0.050
DRS small	1.00	< 0.001*	0.57	0.084
DRS medium	0.82	0.003*	0.47	0.169
DRS big	0.88	< 0.001*	0.49	0.153

### Changes in visual acuity and contrast sensitivity

No significant changes in visual acuity (VA) were observed after training across participants or between groups. This is not surprising since only two of the stereoanomalous participants are amblyopic. Similarly, no significant changes in contrast sensitivity were observed between groups or eye tested for the quick Contrast Sensitivity Function (qCSF) area under the curve or acuity.

### Preliminary control study

Prior to the study, to ensure that Condition 3 of our games required stereopsis for optimal performance, a neurotypical participant played the game under binocular and monocular conditions (via patching). For both games, performance was substantially worse and dichoptic errors increased under the monocular condition (Supplementary Tables S2 and S3). This strongly suggests that the tasks in Condition 3 require stereopsis for optimal performance and cannot be solved otherwise.

### DartBoard in-game performance

The DartBoard game provided 102,252 data points from 20 participants. This resulted in approximately 40 trials per condition and block. On each trial we calculated depth error in arc seconds, which is the difference between the dart landing position and the center of the board.

**Figure 2:**
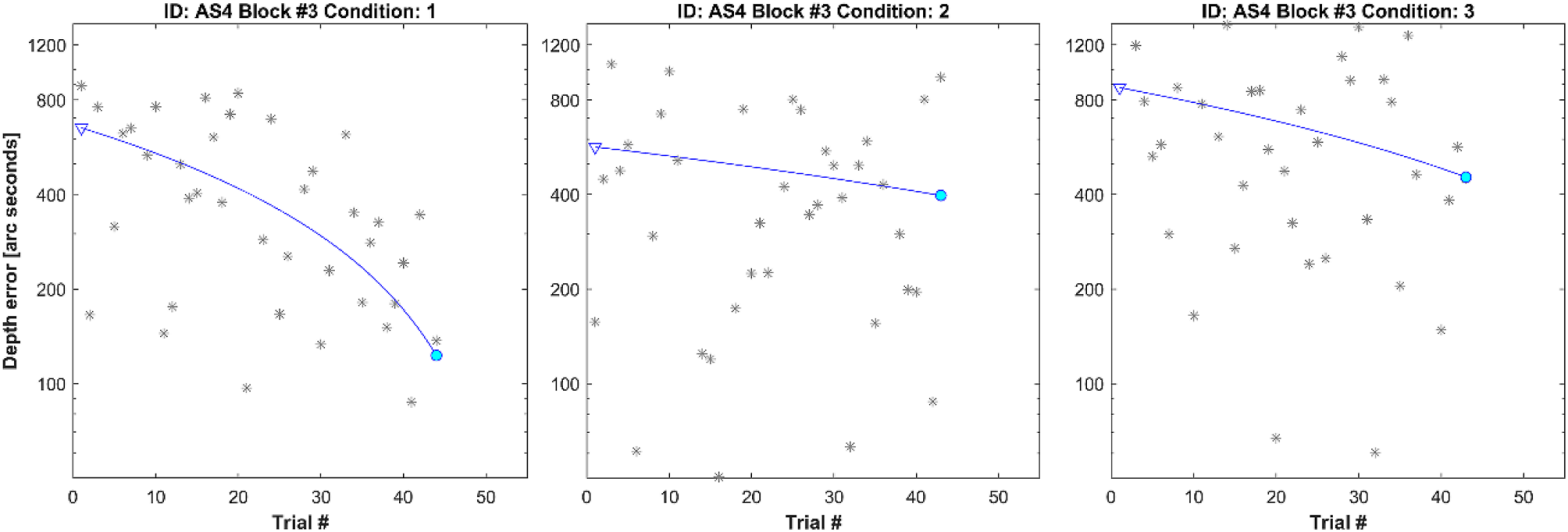
Within-block learning example (AS4, strabismic stereo-anomalous participant). From left to right, raw data in block number 3 under Condition 1, 2 and 3. Each asterisk represents depth error (arc seconds) from one trial. The continuous blue line represents a linear fit of the depth error at each trial. The triangle represents the start point and the circle the end point.

We define within-block learning as a decrease in depth error from the beginning to the end of a particular condition in a specific block. In Fig. 2 we performed a linear fit on depth error per condition and block and extracted three within-block results: initial, final, and mean depth error. Most stereo-anomalous participants exhibit within-block learning per condition.

**Figure 3:**
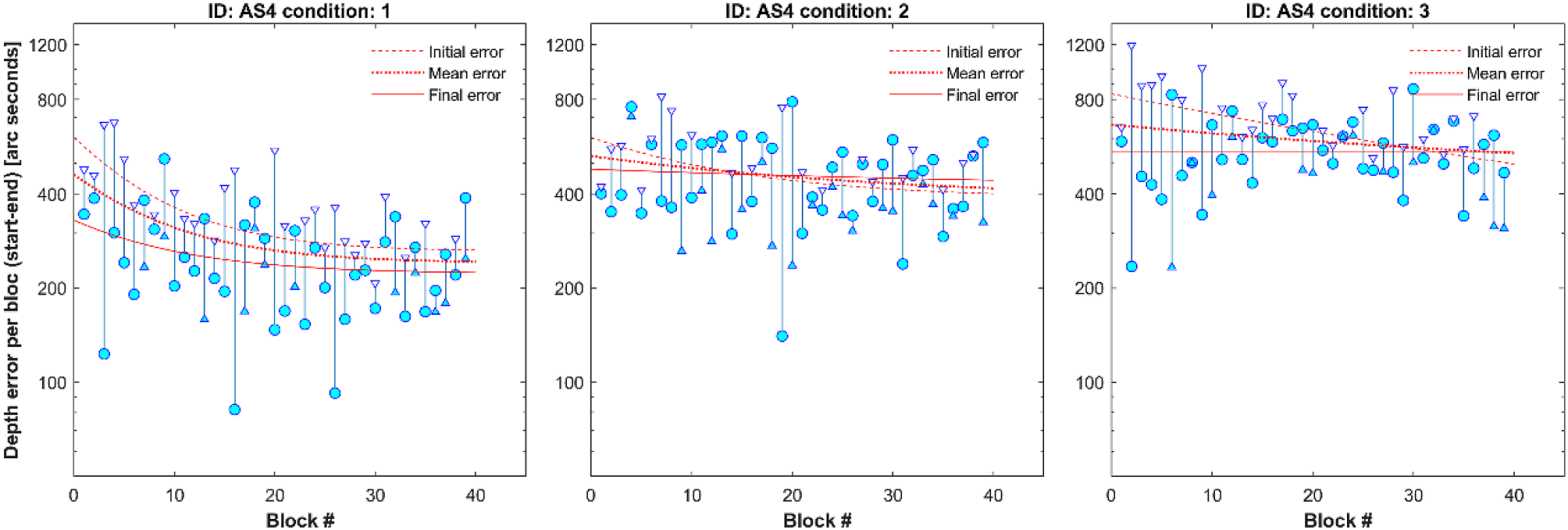
Across-block learning example (AS4, strabismic stereo-anomalous participant). From left to right, results under Condition 1, 2 and 3. Results from each block are represented as a vertical line, with a triangle on one end indicating the depth error at the beginning of the block and a circle indicating the depth error at the end of the block. A triangle at the top indicates depth error reduction within a block. Three exponential plots have been superimposed and represent across-block learning. The fits represent an exponential function to the initial error at each block (dotted line), mean error (hashed line), and final error (continuous line).

After training, an improvement is expected, and we call this across-block learning. Figure 3 shows 40 blocks of DartBoard data obtained from participant AS4 (Supplementary Fig. S1 for others). For clarity, each condition is represented in a different graph. Each within-block result is represented as a vertical line. The triangle represents the initial depth error while the circle represents the final depth error. Blocks with a triangle above the circle indicate a reduction in depth error (i.e., within-block positive learning). To quantify across-block learning, we fit an exponential function to the initial, final and mean within-block depth error for each participant and condition. The difference between the exponential fit of the initial and final depth error for each condition represents the within-block learning trend. AS4 exhibits a positive within-block learning trend in Condition 1, whereas within-block learning in Condition 3 tends to plateau after 30 blocks. The exponential fit also allows us to understand each participants’ learning pattern across blocks. AS4 exhibits a clear across-block positive learning pattern in Condition 1, regardless of depth error considered (initial, mean or final), but not for Condition 2. In Condition 3 only initial depth error exhibits a clear positive learning pattern.

**Figure 4:**
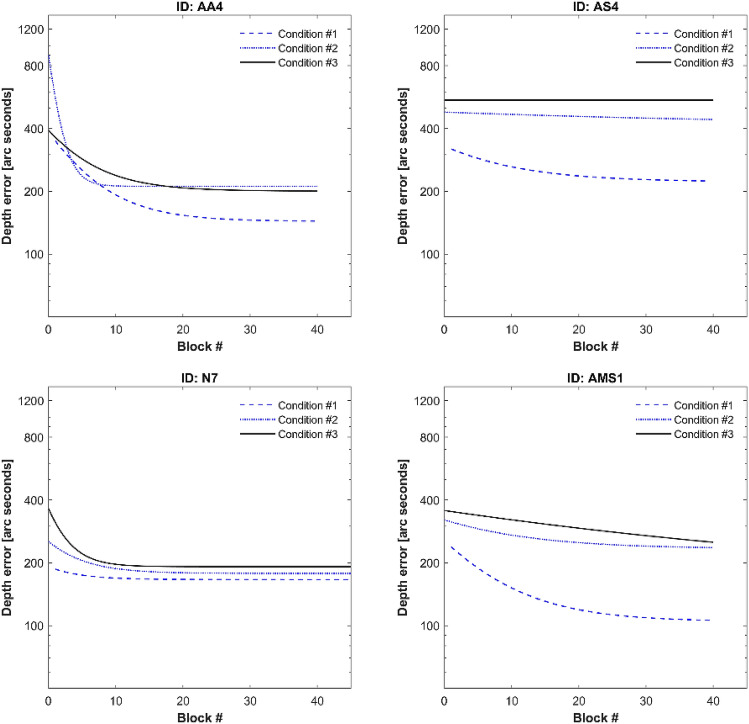
Across-block learning in four participants. From upper left to bottom right: AA4, stereo-anomalous anisometric; AS4, stereo-anomalous strabismic; N7, stereo-normal; AMS1, stereo-anomalous with micro strabismus. Each graph shows the exponential fit of the end-block depth error in the three conditions: Condition 1, blue dashed line; Condition 2, blue dotted line; Condition 3, dark continuous line. Although N7 performed 60 blocks of training, only first 45 blocks are represented to facilitate comparison.

To visualize the across-block learning patterns in greater detail, Fig. 4 shows only the exponential fit using the final within-block depth error for the three conditions in the same graph. This figure compares different learning patterns of four participants (Supplementary Fig. S2 for all participants): AA4 (anisometropic), AS4 (strabismic participant shown in Fig. 3), N7 (stereo-normal) and AMS1 (micro strabismic).

As expected, the final depth error is lower in Condition 1 compared to Condition 3, meaning that it is easier to judge depth when all cues are available. Differences in learning pace and final depth error are more evident in Condition 3 compared to Condition 1. For example, AS4 exhibits no learning in Condition 3, whereas AMS1 has not reached the plateau after 40 blocks. Surprisingly, AMS1 achieved a final depth error in Condition 1 lower than any of these four participants.

### Statistical analyses

To quantify these results, we first analyzed the DartBoard game raw data. Since the raw data did not follow a normal distribution when analyzed as a whole, or considering the six subgroups obtained from pairing participant group and cue scaffolding condition (*P* < 0.01 in all cases), non-parametric tests were used for the following analysis.

Median and interquartile range of trial depth error are shown for each group and condition in Table 3. Stereo-normal participants performed better than stereo-anomalous on each condition. However, Condition 3 provides worse results than Condition 1 or 2 regardless of participant group.

**Table 3:** DartBoard trial depth error median and interquartile range per condition and group.

Condition	Stereo-normal (arc secs)	Stereo-anomalous (arc secs)
1	154 [72–270]	170 [78–308]
2	160 [74–286]	206 [92–377]
3	161 [75–286]	212 [98–393]

Trial depth error differences across conditions are statistically significant for both groups (*P* < 0.001), except between Conditions 2 and 3 for the stereo-normal group (*P* = 0.395). Finally, differences between stereo-normal and stereo-anomalous groups are statistically significant no matter the condition considered (*P* < 0.001).

In a second analysis approach, each participant was characterized by the exponential fit using the final within-block depth error for each condition (Fig. 4). The exponential fit was obtained using a standard exponential function (Eq. 1) with three coefficients (a, b and c) and allows to estimate the depth error (y) for each block (x).

1 $$Y = a - b*e^{{\left( { - c X} \right)}}$$

**Figure 5:**
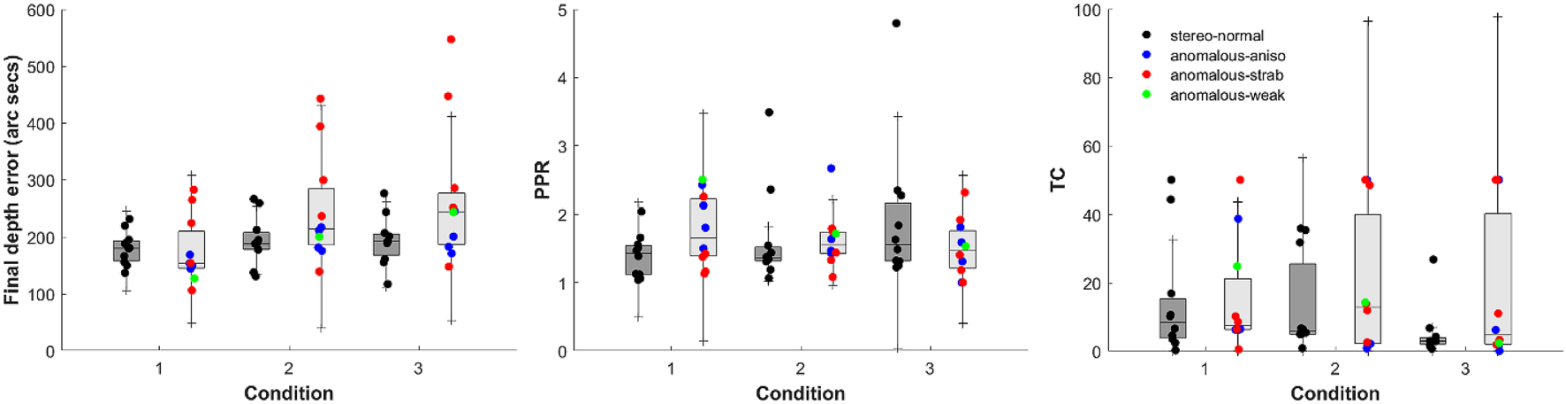
Box plots of DartBoard in-game performance accuracy, from the exponential fits: Final depth error, PPR, and time constant. Medians and interquartile ranges for each group and condition considered. Depth error values in seconds of arc.

Once we obtained the three coefficients, we calculated three variables per participant and condition: the final depth error at the last block (final depth error); the pre:post ratio between the error at the first and the last block (PPR), with a higher PPR indicating greater learning; and the time constant (TC), representing the rate of learning. Mean values and confidence intervals for each parameter per condition and group are shown in Fig. 5.

First, the PPR results show that all participants (except AA2 in condition 3 [PPR = 1]) improved in all cue scaffolding conditions. We also found a statistically significant correlation between the PPR and the initial depth error (but not the final depth error) for the stereo-normal participants in all conditions (Table 4). That correlation is present, although lower, and not statistically significant, for stereo-anomalous participants.

**Table 4:** Pearson’s correlation and *P*-values for PPR and initial stereoacuity threshold for each group and cue scaffolding condition.

Condition	Stereo-normal		Stereo-anomalous	
	Correlation	*P*-value	Correlation	*P*-value
1	0.84	0.002*	0.25	0.494
2	0.81	0.004*	0.35	0.323
3	0.83	0.002*	0.56	0.094

For the stereo-anomalous group, the asymptotic performance appears to be higher (worse) in Condition 3 while the PPR seems to be more dependent on condition, decreasing as participants progressed in the game. Finally, the TC seems to be more dependent on condition, reaching longer values in Condition 3.

Nevertheless, these differences are not statistically significant for any of the three parameters, considering group, condition, or any of the possible combinations.

Shown in Table 5 are the number of blocks (median value) needed to achieve 110% of the asymptotic threshold. Although differences are not statistically significant, the number of blocks it takes for learning to stabilize in Condition 3 is lower compared to Condition 1. Furthermore, the number of blocks it takes for learning to stabilize in the stereo-anomalous group is about twice that of the stereo-normal group.

**Table 5:** Time to achieve learning in blocks (median value). The number of blocks needed to achieve 110% of the asymptotic threshold according to the exponential fit is calculated for each condition and for all participants, stereo-normal group and stereo-anomalous group.

Condition	All participants	Stereo-normal	Stereo-anomalous
1	15.1	12.7	15.1
2	10.0	7.3	14.2
3	5.6	4.9	10.6
All	9.2	7.1	14.2

Finally, we were interested in comparing the improvements in Condition 1, where all depth cues are available and Condition 3, where only retinal disparity is available with the caveat that improvements may depend on the initial error. A participant who exhibits a lower error in Condition 1 but performs poorly in Condition 3, is perhaps likely to improve more in Condition 3 than in Condition 1 (i.e. the lower the initial error ratio between Condition 1 and 3, the lower the PPR ratio between Condition 1 and 3).

**Figure 6:**
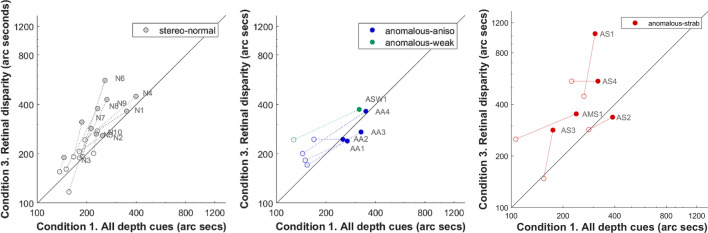
DartBoard in-game performance accuracy initial thresholds and PPR in two cue scaffolding Conditions (1 vs 3) for each group (stereo-normal and stereo-anomalous with strabismic participants plotted separately for less crowding). Each participant is represented as a line, whose start point is a filled circle and end point is an open circle. The start point of the line represents the initial accuracy (arc secs); horizontal line length shows the improvement in game accuracy for Condition 1, and vertical length is the improvement in game accuracy for Condition 3. Points above the diagonal unity line show better performance when all depth cues are present compared to the performance when only retinal disparity is available (as naturally occurs). Lines with angles lower than 45 degrees show greater improvement with all cues than for stereoacuity alone. Stereo-normal participants are represented in gray, stereo-anomalous are represented in different colors depending on subclassification: anisometropic in blue, strabismic in red, stereo-weak in green.

In Fig. 6 each participant is represented as a line, whose start point (filled circle) is the initial error, for Condition 1 on the abscissa and Condition 3 on the ordinate. The length of the horizontal is proportional to the improvement in PPR for Condition 1; the vertical line length is the improvement in PPR for Condition 3. All lines point towards a game accuracy limit that is in the lower left corner of the graph. If performance improves by the same amount in the two conditions, the arrows would be oriented at 45 degrees (parallel to the unity line). Arrows with less than 45 degrees of orientation indicate a greater improvement in Condition 1; arrows with an orientation greater than 45 degrees indicate greater improvement in Condition 3. Stereo-anomalous participants are mainly represented by lines at angles less than 45 degrees, i.e. the improvement attributable to the use of retinal disparity was less than the improvement attributable to the use of all depth cues combined (more similar to natural viewing).

Interestingly, the PPR ratio between Conditions 1 and 3 correlates (Pearson’s test) strongly with initial performance ratio in the stereo-normal group (r = 0.94; *P* < 0.001) but not in the stereo-anomalous group (r = 0.53; *P* = 0.117). Worse initial performance in Condition 1 compared to Condition 3 in stereo-normal participants predicts greater improvement in Condition 1 after treatment compared to Condition 3, but this is not necessarily true for stereo-anomalous participants. Similarly, this happens if performance in Condition 3 is worse than in Condition 1. We understand that the treatment benefits are more evident for the weaker initial condition in stereo-normal participants, but this trend is not clear for stereo-anomalous participants.

### Halloween in-game performance

Our in-game performance measures for Halloween yield the proportion of correct responses (hits) for each stereoscopic demand (1000, 800, 600, and 400 arc secs) and each cue scaffolding condition for each session. To assess improvements, we performed an m-alternative signal detection (d’) analysis^44^, since the number of choices varied across trials (from 3 to 7). This analysis takes into account the number of available choices (targets) at each stereo demand^45,46^. Specifically, we computed d’ for the first three hours of game play (pre) and the last three hours (post) to get a PPR assessment. Supplementary Figure S3 shows that most participants improved their accuracy in the last three hours compared to the first three hours with the largest and smallest disparity levels across the three conditions.

**Figure 7:**
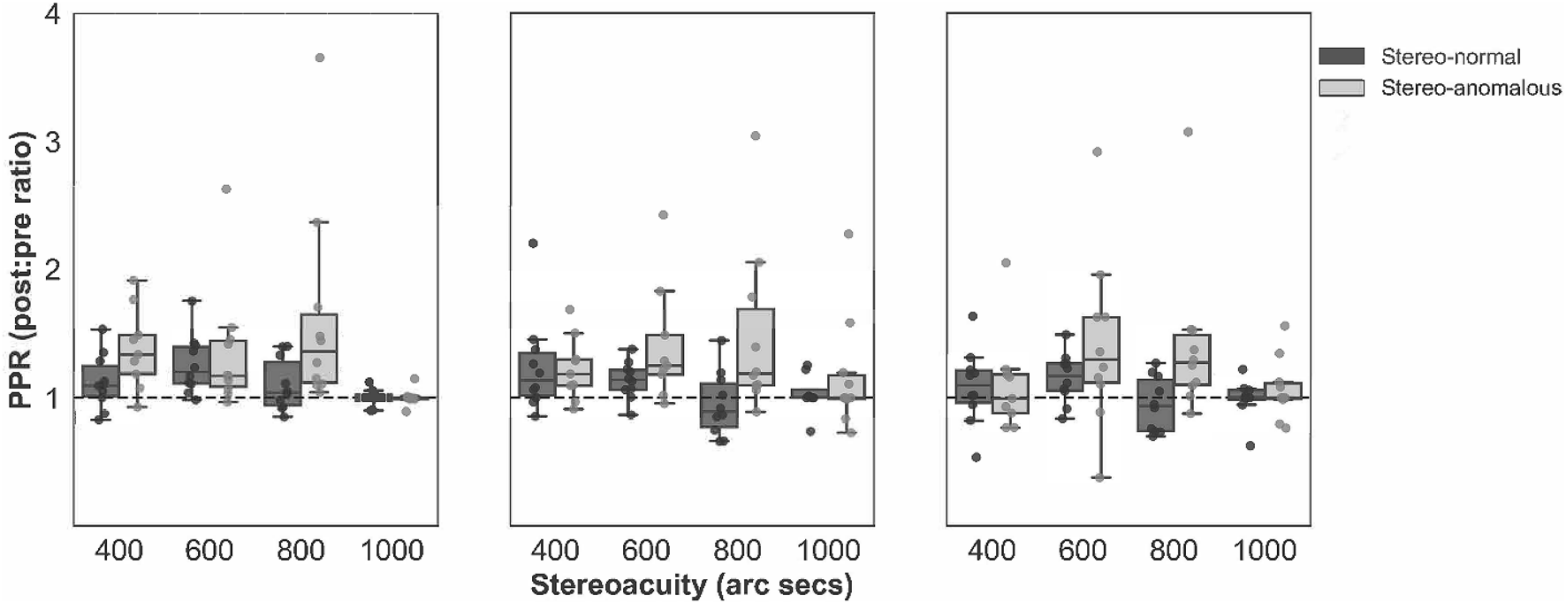
Box plot comparing d’ PPR values between stereo-normal (dark grey) and stereo-anomalous (light grey) groups across stereoacuity demand (400″, 600″, 800″, and 1000″) and Conditions (1, 2, and 3). Condition 1 (left panel), Condition 2 (middle panel), and Condition 3 (right panel). Each symbol represents individual data.

To assess the amount of improvement, we took the PPR of the d’ value for each participant, cue scaffolding condition, and stereoacuity demand (Fig. 7). Our analysis revealed a statistically significant difference between groups in d’ PPR for Condition 1 (*P* = 0.048) and Condition 2 (*P* = 0.035), but not for Condition 3 (*P* = 0.100) (Table 6). Indicating that the stereo-anomalous group increased their sensitivity to detect the stimulus more than the stereo-normal group. However, only d’ PPR for 800″ was statistically significant between the groups (stereo-normal *M* = 1.0; stereo-anomalous *M* = 1.51, *P* < 0.001). When comparing groups across cue scaffolding condition and stereoacuity demand, there was a significant difference in d’ PPR for Condition 1 and 800″ (*P* = 0.019), Condition 2 and 800″ (*P* = 0.015), and Condition 3 and 800″ (*P* = 0.012), but not between the remaining combinations (Table 7).

**Table 6:** Kruskal–Wallis mean comparisons between group and condition for PPR d’ values.

Condition	Stereo-normal	Stereo-anomalous	*P*-value
1	1.128	1.344	0.049*
2	1.094	1.322	0.035*
3	1.049	1.258	0.100

**Table 7:** Mann–Whitney pairwise comparisons between group, condition, and disparity demand for PPR d’ values.

Condition	Disparity	Stereo-normal	Stereo-anomalous	*P*-value
1	1000	0.99	1.00	0.675
	800	1.10	1.63	0.019*
	600	1.26	1.36	0.912
	400	1.13	1.38	0.095
2	1000	1.03	1.17	0.856
	800	0.95	1.48	0.015*
	600	1.14	1.41	0.156
	400	1.25	1.23	0.720
3	1000	0.99	1.07	0.822
	800	0.95	1.42	0.012*
	600	1.16	1.43	0.280
	400	1.09	1.11	0.604

**Figure 8:**
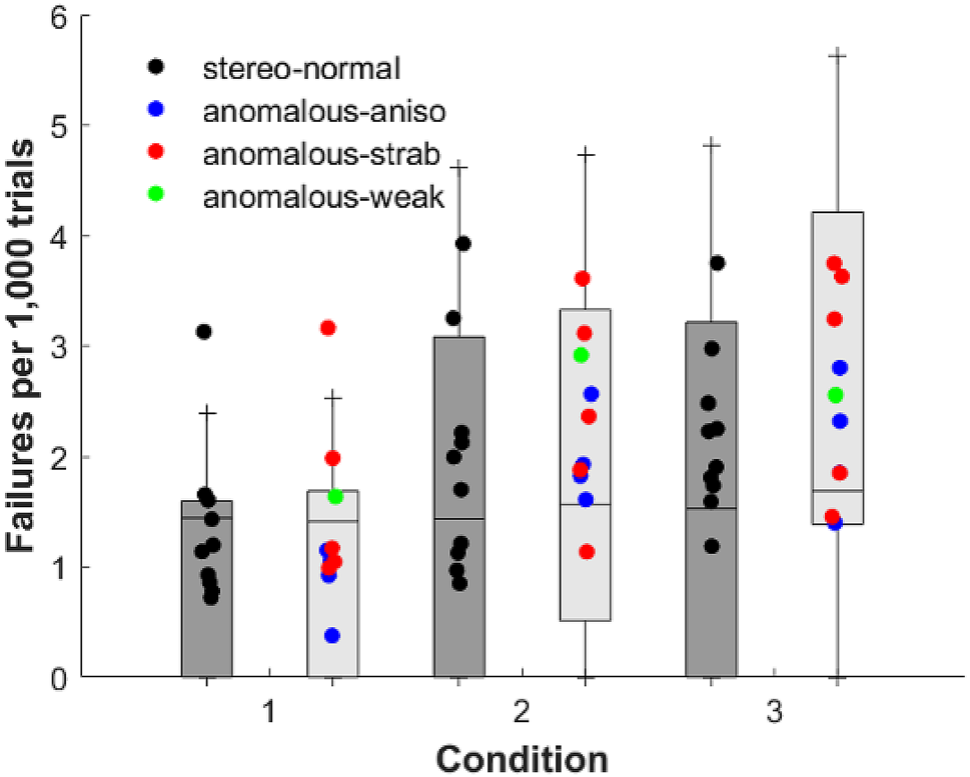
Box plots of Halloween game failures to detect dichoptic targets per 1,000 trials for stereo-normal (grey bars) and stereo-anomalous (white bars) groups for each Condition (1, 2 and 3). Symbols represents data from one participant: stereo-normal (black), anisometropic (blue), strabismic (red) and stereo-weak (green). The horizontal line represents the group median while the whiskers represent the interquartile ranges.

The suppression task, inserted in the Halloween mechanics, proved to be valuable as a means of tracking participant engagement and suppression episodes. Figure 8 shows each participants’ failure to detect one of the dichoptic targets.

A Kolmogorov–Smirnov test showed that the dichoptic errors did not follow a normal distribution when analyzed as a whole or when considering the six subgroup pairings between participant group and cue scaffolding conditions (*P* < 0.001 in all cases), thus a Kruskal–Wallis test was applied. Our analysis revealed significant differences as a function of condition for the stereo-normal (*P* = 0.006) and stereo-anomalous group (*P* < 0.001), except between Condition 2 and 3 in the stereo-anomalous group (*P* = 0.330). Furthermore, Mann–Whitney pairwise comparisons showed significant differences between the stereo-normal and stereo-anomalous group in dichoptic errors for all conditions when considered as a whole (*P* = 0.041) and in Condition 2 (*P* = 0.023), but not in Condition 1 (*P* = 0.578) or Condition 3 (*P* = 0.109).

## Discussion

Our aim was to evaluate whether cue scaffolding and dichoptic PL in VR could be used as a platform to train stereovision. For this proof of concept study, we designed two custom video games, which use a combination of demanding stereovision tasks. Our results show that most stereo-anomalous participants improved in the games and most importantly, the learning transferred to clinical and psychophysical stereoacuity tests (Fig. 1). Despite the different design and nature of the video games, these results support the viability of training stereoacuity by means of videogames, as other studies have previously shown^24,29,30,39^.

Our small sample size of participants with anisometropia or strabismus does not allow us to make statistical inferences beyond the stereo-normal and stereo-anomalous groups. Nevertheless, some participants did not show improvement across stereoacuity tests (AS1, AS2, AA3, and ASM1); all with strabismus except for AA3 who has anisometropia. It has been well documented in the literature that persons with anisometropia retain better stereoacuity at low spatial frequencies^25^. Although their stereoacuity is not as acute as normal, it is nevertheless functional. Therefore, individuals with anisometropia are more likely to recover stereoacuity after treatment.

On the other hand, stereoacuity in people with strabismus is more impaired^24^. A possible explanation for this difference may be that in order to avoid diplopia, suppression scotomas may be playing an active role in strabismus, whereas in anisometropia, suppression may be playing a more passive role, as a result of degraded visual acuity.

Furthermore, people with strabismus have been shown to be more resistant to stereoacuity training compared to people with anisometropia^24,29^. Indeed, participants AMS1, AS1 and AS2 (all strabismic) showed no improvement when measured with RD3, but showed improvement with the other stereoacuity tests. This may be due to poor performance on tests with random dot stimuli in people with subtle binocular angles of deviation. Participant AMS1, with micro-strabismus, is likely to have developed a harmonious anomalous correspondence^47^, providing some binocularity. Training stereopsis cannot succeed in the absence of some neural substrate for binocular fusion.

Regarding the stereo-normal group, most showed small improvements in the psychophysical stereoacuity tests. The small improvements are likely due to a test ceiling of 20 arc seconds. Those improvements were not detected by the clinical tests since all stereo-normal participants were at ceiling at the beginning of the study. However, previous studies have shown improvements in stereoacuity for individuals with normal binocular vision after training^48^ or playing 3D (but not 2D) videogames with large disparities^41^. Furthermore, in certain professions, where specific stereo demanding tasks are common such as those required from dressmakers, stereoacuity seems to be enhanced^49^. There is reasonable doubt about whether good stereoacuity is a requirement for becoming a dressmaker or whether stereoacuity is enhanced by continuous stereo demanding tasks. However, our results indicate that training can improve stereoacuity in individuals with normal binocular vision.

As for VA, we did not detect changes after training, which is not surprising, since only two of our participants were amblyopic. Previous studies aimed at improving stereoacuity have reported a lack of change in VA after training^29,30,48^. Furthermore, current state of the art of VR headsets lack fine resolution, which makes them a poor tool for VA training. Similarly, we did not find significant changes in contrast sensitivity.

For both clinical and psychophysical stereoacuity tests, stereo-normal participants with worse initial stereoacuity thresholds show a higher PPR, i.e., greater improvement (Table 2). DartBoard in-game results show the same trend (Table 3). Somewhat surprisingly, the same is not reflected in the stereo-anomalous group with non-significant correlations. A recent study using a PL stereo training paradigm with random dot stimuli in participants with a history of amblyopia found a strong inverse association between initial stereoacuity threshold and PPR^50^. Similar trends have also been reported for VA recovery, showing that baseline acuity loss does not predict PPR after dichoptic training^51^.

For participants with strabismus, no change was detected in visual angle deviation. There is strong scientific evidence for the success of training convergence insufficiency^52^. Despite each game requiring participants to diverge (DartBoard) or converge (Halloween) to moving targets, the small sample in our proof of concept study is not sufficient to detect changes if they occurred.

Although participants did not adjust luminance balance once set, the Halloween game recorded failures to detect dichoptic targets, where participants were either suppressing or unaware of the task. Stereo-normal participants are not expected to have problems with suppression. However, they exhibit a statistically significant increasing rate of failures across conditions, which may be attributed to binocular rivalry in the headset, or more likely, boredom or fatigue. Stereo-anomalous participants however, behave differently, with a higher error rate, i.e. the dichoptic errors are not only a measurement of fatigue but of something else, likely suppression or rivalry. Nevertheless, embedding a binocular imbalance test in a VR device^53^ seems worthwhile. This would allow researchers the ability to track binocular vision beyond stereoacuity function. Anti-suppression therapy by means of dichoptic games has little or no effect on stereoacuity according to previous studies^39,54–56^. However, direct stimulation of stereoacuity does appear to contribute to the re-balancing of binocular vision by reducing suppression^48^. Indeed, it has been suggested that improved stereoacuity after PL might reflect a decrease of interocular suppression^30^, although it could also be the result of a signal enhancement in the amblyopic eye.

Although gamification can be a useful tool for increasing motivation, attention and compliance, it comes at a cost. Game results are not as sensitive in tracking the participants’ evolution compared to results obtained through traditional PL tasks. Nevertheless, the DartBoard game results are coherent with clinical and psychophysical tests. First, trial depth error, regardless of the condition, differentiates stereo-normal and stereo-anomalous participants. Second, improvement in Condition 3 (only retinal disparity available), is evident in all participants with the exception of one stereo-normal participant (PPR > 1).

The nature of the Halloween game does not provide as rich a dataset as Dartboard. The disparities used in the game were large, with the lowest stereoacuity demand set to 400″. Given these conditions, it is reasonable to suggest that perceptual training took place at or slightly above threshold for most stereo-anomalous participants, whereas clearly above threshold for stereo-normal participants. Nevertheless, stereo-normal participants also improved. When designing a PL task, above-threshold activities are not considered since there’s a notion that the activity would become (even) less interesting and engaging^24^. Nevertheless, at least one study has reported improvements in stereoacuity in a control group whose activity was chosen to be above threshold^40^. In that case the improvement was attributed to the stimuli used (random dots), which potentially improved binocular fusion and signal to noise discrimination. Those aspects may be especially important for strabismic patients. We cannot definitively know whether the improvement in clinical and psychophysical measures of stereoacuity were due to DartBoard and/or Halloween, but it is important to point out that strabismic observers fare better with larger disparities^57^. Thus, the Halloween stimulus might have allowed them to strengthen their stereopsis by providing a stimulus they can latch on to.

Finally, the novelty of this study is the use of a cue scaffolding approach for improving stereovision. We demonstrated that cue scaffolding is present using DartBoard results: trial depth error differences across conditions are statistically significant regardless of group assignment (except Condition 2 and 3 for the stereo-normal group). Differences are more notable between Condition 1 (almost all depth cues available) and Condition 3 (only retinal disparity).

When we analyze DartBoard within-block learning, we observe that in most blocks there is a positive difference between initial and final depth error, meaning that the participant’s skill improves during the block on any condition (Figs. 2 and 3). This behavior is not present in all blocks (Fig. 3), maybe due to fatigue, and because of the nature of the proof of concept study the trend does not reach statistical significance. In any case, within-block learning also seems to be more evident in the first blocks of training than in the last (Fig. 4), meaning that after some training participant responses are more consistent during a new block practice. A key feature of cue scaffolding is that improvements made in the previous condition potentially influence the depth error of the condition that follows. We detect this behavior when surprisingly, the final depth error in Condition 3 is lower than in Condition 1 in one block (Supplementary Table S2, depth error under binocular vision results). How is it possible that the performance is better when all depth cues have been removed except binocular parallax? The logical explanation is that performance on a condition is influenced by the previous condition. This approach can be especially important for patients with strabismus and/or poor baseline stereoacuity, who might benefit from a design where associations between monocular and binocular cues are strengthened over time. Beginning each training session with the easiest condition where all binocular cues to depth are available and progressing to the last condition where disparity is the most reliable cue to depth is analogous to starting each session with training wheels and removing them at the end.

Stereo-normal participants tend to improve more in conditions with worse performance (Fig. 6). However, stereo-anomalous participants show an improved ability to integrate all depth cues. Previous studies^48^ have demonstrated that in adults deprived of normal binocular vision, repetitive depth demanding tasks contribute to a reweighting of depth cue integration where the weight of the disparity cue is increased (learned behavior). However, they do not all achieve the same reweighting as normal control participants. In situations where disparity depth valuation is contradictory with other depth cues, e.g., texture, the weight of disparity in the final estimation increases after treatment. Sensory integration (Condition 1) is less resistant to improvements than just disparity depth perception (Condition 3). This might explain why patient reports of improved depth perception after visual therapy treatments are not correlated with measurable stereoacuity improvements^58^.

Our interventional model of direct stereopsis stimulation using VR and incorporating depth cue scaffolding improved in-game performance in normal and stereo-deficient subjects. This improvement transferred to stereoacuity measured with both clinical and psychophysical stereoacuity tests. Importantly, this approach provides rich in-game performance measures which may provide useful insights into principles for effective treatment of stereo anomalies.

## Methods

### Participants

Twenty adults (mean age: 28 ± 2.5, range: 18–62 years, 14 female), 10 with normal or corrected-to-normal vision and without ocular pathologies (stereo-normal group) and 10 with binocular impairment (stereo-anomalous group), participated in the study. Participants were recruited by telephone from the Meredith W. Morgan University Eye Center’s internal list of patients who gave written consent to be contacted for research studies and through internal UC Berkeley student list serves. The Institutional Review Board of the University of California, Berkeley approved the study protocol. The study was conducted according to the tenants of the Declaration of Helsinki and informed consent was obtained from each participant. Exclusion criteria for the study included: (1) ocular pathologies (e.g., macular abnormalities) or nystagmus, (2) non-concomitant or large angle constant strabismus (> 30 prism diopter), (3) inability to fuse, (4) constant esotropia (> 20 prism diopters), (5) VA ≥ 20/200, and (6) previous dichoptic visual training of more than 10 h.

### Study design and training

**Figure 9:**
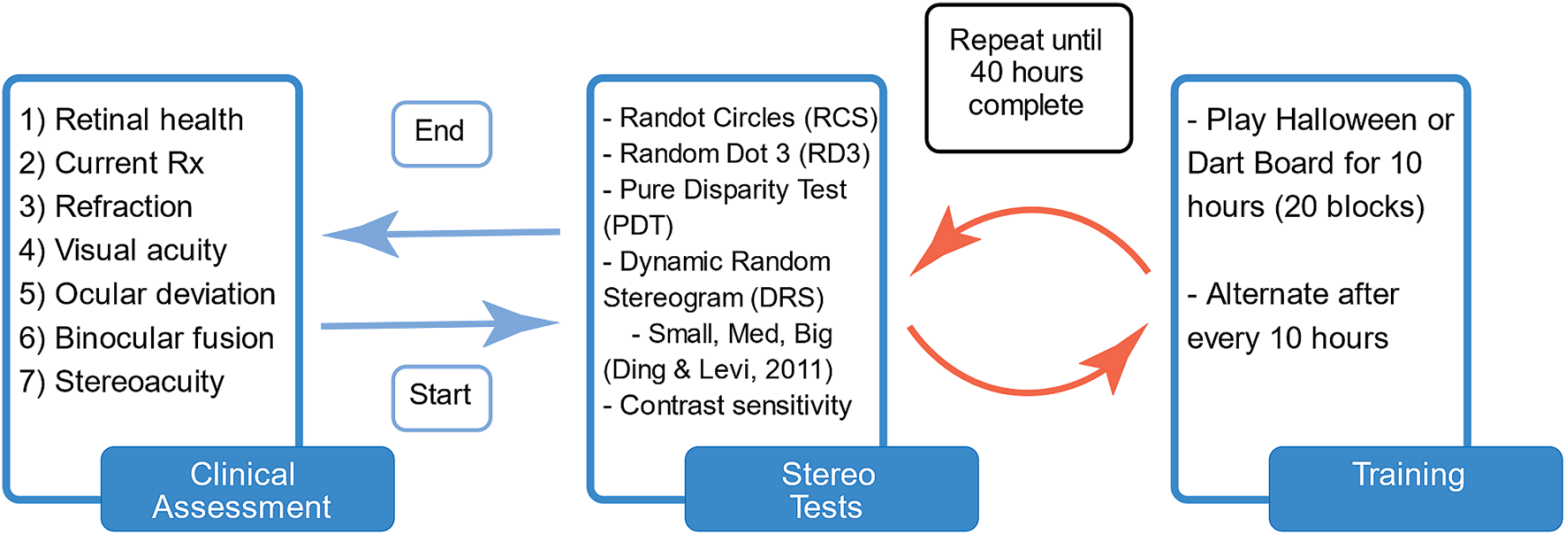
Study and training schematic. Each participant began with a clinical assessment. Followed by clinical and psychophysical stereoacuity tests. Participants then alternated between playing one of two games (Halloween or DartBoard) for 10 h. After every 10 h (20 blocks), clinical and psychophysical stereoacuity tests where administered until 40 h were completed. Lastly, the clinical assessment was administered.

All participants underwent a complete clinical assessment before and after the study (Fig. 9). The complete clinical assessment included evaluation of: (1) retinal health (ophthalmoscopy), (2) current prescription, (3) refraction at distance, (4) VA (Bailey Lovey visual acuity chart), (5) ocular deviation (monocular cover-uncover test and alternate cover test, using accommodative stimuli, and 4BO test), (6) binocular fusion (Worth Dot at 33 cm and 3 m) and (7) clinical stereoacuity (Randot Circles Stereotest and Random Dot 3 Stereo Acuity Test with Lea Symbols).

Participants were categorized as having anisometropia if there was a difference ≥ 0.50 D in spherical equivalent refraction or ≥ 1.5 D difference in astigmatism in any meridian, between the two eyes^59^. Participants were classified as having strabismus in the presence of a tropia with the cover test and/or showing micro-strabismus by the 4^∆^BO test.

Following the clinical assessment, eligible participants were placed in either the stereo-normal or stereo-anomalous group based on their initial Randot Circles Stereotest stereoacuity measurement. Inclusion criteria for the stereo-anomalous group was baseline stereoacuity of 50 arc secs or worse. According to this criterion, ten participants were assigned to the stereo-anomalous group and ten to the stereo-normal group. Four participants in the stereo-anomalous group had anisometropia and five strabismus (one of them micro-strabismus). The remaining participant in the stereo-anomalous group did not exhibit anisometropia or strabismus and was labeled stereo-weak (Supplementary Table S1).

Training was organized in four 10-h intervals, where participants played one of the two games designed specifically for this study: Halloween or DartBoard. On each day of training, participants played two 30-min blocks for a total of one hour. Most participants completed 80 blocks of training with the exception of N2 and N4 who completed 40 blocks, and AS1, AA1, N5, N7, N9, N10 who completed 100 blocks total (in these cases, DartBoard was played for 60 blocks).

After every 10 h of game play, participants completed the clinical and psychophysical stereoacuity tests. For clinical stereoacuity, we used the random-dot stereograms RCS and RD3. For psychophysical tests, we used the PDT and DRS described in detail in Ding & Levi, 2011. Briefly, stimuli were viewed through a stereoscope and presented on a Sony CRT monitor (CPD-G500) at a viewing distance of 68 cm from the participant. To correct for misalignment, participants were shown a nonius cross with a fusion-lock frame and were able to move the position of the mirrors to align the cross. For participants with strabismus, added prisms were used if necessary. DRS stimuli consisted of circular bright dots (126 cd/m^2^) on a dark background (1.37 cd/m^2^) and were presented in three different sizes (small: 22.64, medium: 90.55, big: 362 arc secs). PDT stimuli consisted of two 3° × 3°sine-wave gratings (0.67 cpd) at 48% contrast with sharp edges. DRS and PDT stimuli were visible to the participant for 2 s.

In addition to stereoacuity, we monitored contrast sensitivity using the qCSF with a Bayesian staircase^60^. The qCSF test was displayed on a 46″ NEC LCD monitor (model p463) with a resolution of 1920 × 1080 and a contrast ratio of 4,000:1. Participants were seated in a chair 6 m from the screen. CSF was measured on the dominant (DE), non-dominant (NDE), and both eyes (OU). For the stereo-anomalous group, NDE was determined by amblyopic eye in participants with amblyopia, deviated eye in participants with strabismus, and eye with worse VA in participants with anisometropia. For the stereo-normal group, eye dominance was assigned at random. The qCSF test consisted of 25 trials for each eye condition. On each trial, participants were presented with a set of three letters of the same size in decreasing spatial frequency and luminance from right to left. Participants were instructed to identify the letters on the screen.

Once the training was considered complete, the clinical assessment was administered again.

### Games and apparatus

The games were played using the Oculus Rift DK-2, which is equipped with a gyroscope, accelerometer, and a magnetometer with an update rate of 1000 Hz. The Oculus Rift DK-2 has a resolution of 960 × 1080 for each eye, a 100-degree field of view, a refresh rate of 60–75 Hz, and a position tracking refresh rate of 60 Hz. To run the software, we used the Alienware AREA51R2 computer with Intel Core i7-5820 K CPU and an NVIDIA GeForce GTX 980 graphics card.

**Figure 10:**
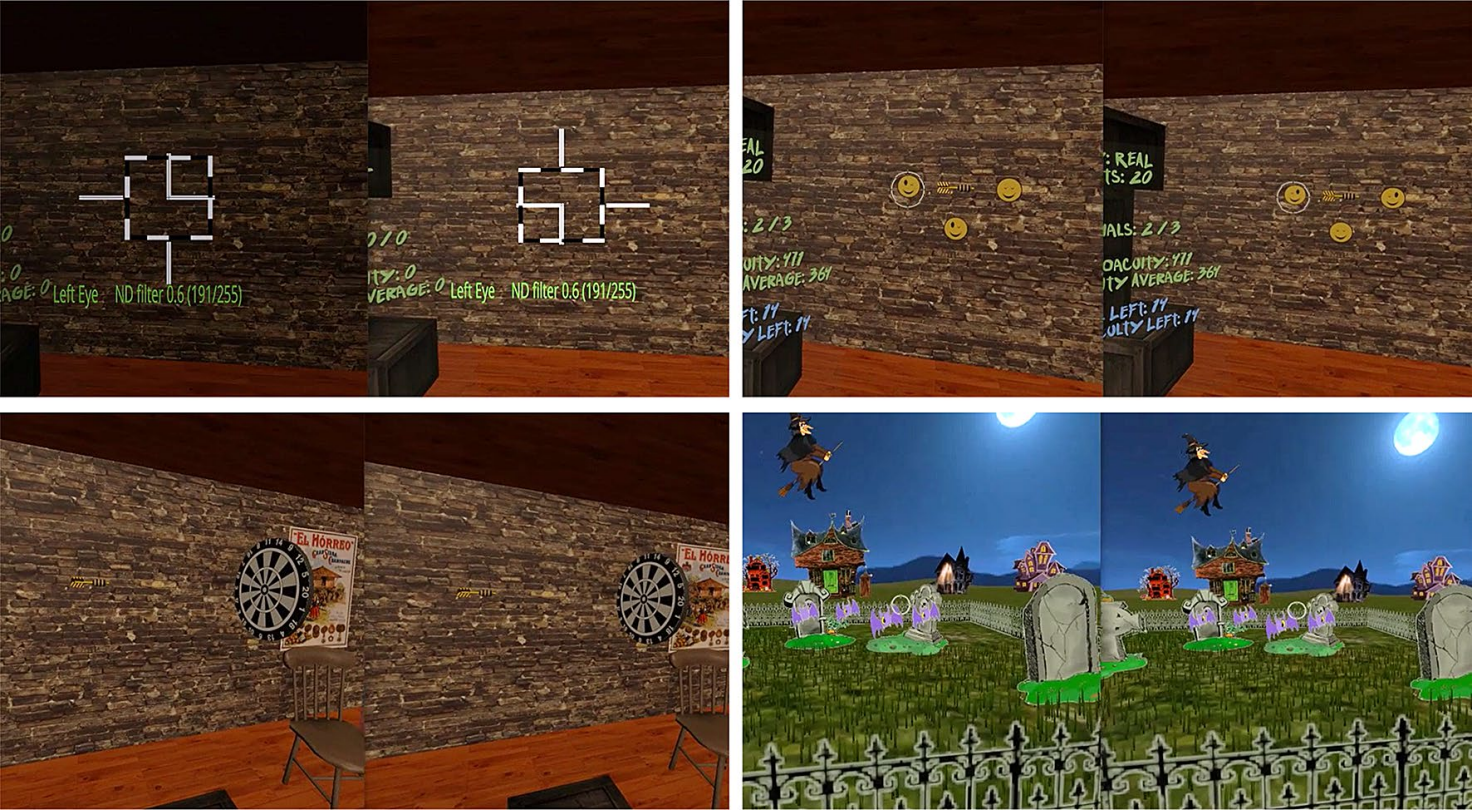
DartBoard and Halloween game screenshots. Top left: Fusion-lock frame calibration for DartBoard (similar in Halloween) to eliminate subjective misalignment angles. Top right: DartBoard 3-AFC suppression task. Bottom left: DartBoard trial example. Bottom right: Halloween trial example.

Importantly, for participants who were unable to fuse the images due to strabismus and/or suppression, games started with a high contrast fusion-lock frame presented to each eye, and a dichoptic nonius calibration. To correct for misalignment, the researcher manually adjusted the images presented to each eye (horizontal, vertical, and cyclo deviations, plus aniseikonia) until the participant reported complete alignment of the dichoptic cross (Fig. 10- Top left), allowing correction for any deviation in the subjective angle of squint. To minimize or eliminate suppression, image luminance (ranging from equal luminance of both images to complete occlusion of one eye) was adjusted for the dominant eye until participants reported equal luminance of the dichoptic lines crossing at the reference frame.

Briefly, the Dartboard game required participants to judge the movement of a dartboard in depth (z-direction) and launch a dart (presented in front, perpendicular to the participants’ eyesight) to hit the center of the board. After each attempt, they were given an auditory tone to indicate when they hit the board and visual feedback indicating the number of points they received. If the dart hit the center of the board, participants received a trophy which was displayed on the screen. Additionally, a scoreboard to the left of the participant kept track of a number of performance variables (e.g., condition, points, accuracy (stereoacuity in the video), average accuracy).

The main variables in the Dartboard game are the dart and the back wall, which are at a distance of 1.5 and 2.5 m respectively, from the participant. The board movement begins linearly and perpendicular to the wall, leaving a gap from the dart (0.7 m), which is the third variable. Movement of the board is paused at certain time intervals (pause of 0.5 ± 0.1 secs each 1.0 ± 0.2 secs of movement) to facilitate estimation of depth. However, to avoid any time-learning effects, both the dart and the board-to-dart gap distance change randomly from trial to trial (dart distance 1.5 ± 0.5 m, board-to-dart gap 0.7 ± 0.2 m). Furthermore, the speed of the board also changes between trials (0.25 ± 0.02 m/sec) and a perpendicular movement in the direction of the dart is also introduced (± 0.05 m/sec). Finally, as we will explain shortly, to avoid perspective cues in Condition 2 and 3, the size of the dart (length 0.20 ± 0.04 m) and the board (diameter 0.40 ± 0.08 m) vary from trial to trial.

The Halloween game required participants to judge which target in a series of 3–7 was closest and eliminate them sequentially (see video for reference). Again, participants were presented with both auditory (gunshot sound) and visual (points and written feedback such as “Great!”) to indicate that they hit the target.

In the Halloween game, targets (phantoms, vampires, pumpkins) are presented at 2 m from the participant (first scene variable) and move closer to the participant at time constant intervals (5–8 steps, each 4 secs long), until they reach a close distance point (1.5 m from the participant). The player’s task is to destroy the targets before they reach the close distance point. All targets (e.g., phantoms) are at different distances and before they reach the close point, the player must destroy them in order from closest to farthest. One of the targets presented can be an anti-suppression target named cyclops (has only one eye and is presented to only one eye of the participant), which should be avoided (i.e., not destroyed). The game is organized in levels, where the number of targets and steps varies (e.g., in level 1 there are five steps and three phantoms; in level 4 there are four phantoms plus one cyclops and eight steps). Linked to the concept of level is the stereoacuity demand (range: 800–400), i.e., the depth difference between each two consecutive targets: as an example, the depth between targets in level 1 is 800 arc secs while in level 4 the depth is 400 arc secs.

Cue scaffolding was implemented in both games, creating three consecutive cue scaffolding conditions (Condition 1, 2, and 3), which progressively minimized or eliminated non-stereoscopic depth cues, from an up-to-date VR scene (where accommodation was the only depth cue not simulated) to a scene where only retinal disparity was available. Each block began with Condition 1, which consisted of non-binocular and binocular cues to depth including shadows, perspective, motion parallax, and binocular disparity. In Condition 2, shadows were eliminated and perspective reduced by removing relative size as a reliable cue (i.e., object size was not relative to object distance). Lastly, in Condition 3, motion parallax was limited (only rotational movements of the head were allowed by the software), making binocular disparity the most helpful (almost unique) cue to calculate distance.

A suppression task (by means of dichoptic images) was also inserted in each games’ mechanics to help participants become aware of suppression episodes. For example, in the DartBoard game, participants were instructed to identify the smiley face (in a set of three) with both eyes open (3-AFC), which could only be seen if binocular fusion was maintained (Fig. 10- Top right). In the Halloween game, participants were instructed to destroy all targets with both eyes open and avoid targets with only one eye, which again could only be achieved if binocular fusion was maintained. Importantly, the purpose of the suppression task was to bring awareness of suppression episodes to participants who actively suppressed. Suppression failures were registered in both games.

Game mechanics and respective in-game measurements were different in the two games. In DartBoard, participants were instructed to launch a dart, which was presented at the center of the screen, towards a dartboard that traveled from the back of the participant and moved towards the background of the scene (Fig. 10- Bottom left). Each dart launch ended a trial. Movement of the dart once launched, was always linear and traveled from left to right, while movement of the board was linear, but not necessarily at a 90° angle or at the same speed of the dart. Both linear movements occurred in the same plane where the observers’ eyes were. Thus, there was no way of guessing the intersection of both trajectories using purely monocular cues. The motion of the dart board was designed to stop at intervals in order to facilitate the exercise. The perceptual learning task for the participant was to estimate, using the background wall as reference, when the dart (the stationary object) and the dart board (the object moving in depth) were at the same distance (i.e. depth error could theoretically be as low as zero arc secs).

In Halloween, participants were instructed to shoot the closest target in a variable set of targets (from three to seven phantoms, pumpkins or vampires) as they approached the participant (Fig. 10- Bottom right). Each shot ended a trial. Similar to the movement of the dartboard, the approaching targets stopped at intervals to facilitate the exercise. Importantly, relative parallax between the targets were constant, and decreased across trials from 1000″ to 400″ over time, depending on the participants’ performance. Therefore, the perceptual learning task in Halloween was to determine the *relative depth distance* between several objects (an n-alternative forced choice task). It is important to note that DartBoard in-game measurements result in depth error values for each trial and for each cue scaffolding condition, while Halloween in-game measurements result in the proportion of correct responses for each stereo demand at each condition.

### Data analysis

Differences between stereo-anomalous and stereo-normal groups and/or cue scaffolding conditions were calculated through mean comparisons. Analysis of variance was used to establish differences between variables with more than two levels of comparisons. ANOVA was used for variables with a normal distribution and Kruskal–Wallis when distributions were not normal. We performed a two-sample comparison using the Student’s t-test when data followed a normal distribution and Wilcoxon-Mann–Whitney otherwise. The Kolmogoro-Simirnov test was used to confirm normal distribution of data. The relationship between variables was made through Pearson’s correlation. The significance level was set to 0.05 for all comparisons. The R-Statistics (v3.6.3) and Python (3.6.8) were used to run the analysis.

## Supplementary Information

Supplementary Information 1.

Supplementary Video 1.
