# Supplementary material for: Scaffolding depth cues and perceptual learning in VR to train stereovision: a proof of concept pilot study
Source: Sci Rep. 2021 May 12;11:10129. doi: 10.1038/s41598-021-89064-z (PMC8114935; doi:10.1038/s41598-021-89064-z)
Supplement: Supplementary file 1 — Supplementary Information 1. [file 41598_2021_89064_MOESM1_ESM.pdf]

Scaffolding depth cues and perceptual learning in VR to train stereovision: A proof of concept pilot study.

Angelica Godinez[1,*], Santiago Martín-González[2], Oliver Ibarrondo[3], Dennis M Levi[1]

[1]School of Optometry, University of California, Berkeley
[2]Construcción e Ingeniería de Fabricación, Universidad de Oviedo, Spain
[3]OSI Alto Deba, Unidad de Investigación AP, Arrasate, Spain
*angelica_godinez@berkeley.edu

SUPLEMENTARY MATERIAL

Table S1

Participant clinical details.

SID	Age, Gender	Dx	Visual acuity	Ocular alignment	Refractive error	Fixation
**AA1**	24, M	Aniso	OD: 20/16<br>OS: 20/25 +1	Far: ortho<br>Near: 4 EP	OD: +1.25 -1.00 x002<br>OS: +4.25 -1.50 x170	Central
**AA2**	18, F	Aniso	OD: 20/25 -1<br>OS: 20/20 +1	Far: ortho<br>Near: ortho	OD: +1.25 -1.25 x188<br>OS: +0.25 -0.25 x009	Central
**AA3**	25, F	Aniso	OD: 20/20 +1<br>OS: 20/16	Far: 4 EP<br>Near: 5 EP	OD: +3.00<br>OS: plano	Central
**AA4***	62, M	Aniso	OD: 20/40 -2<br>OS: 20/20 -1	Far: ortho<br>Near: ortho	OD: +7.75 -2.25 x085<br>(add 2.25)<br>OS: +5.25 -1.50 x105<br>(add 2.25)	Central
**AMS1***	21, M	Strab	OD: 20/25 -2<br>OS: 20/50 -2	Far: ortho<br>Near: ortho	OD: +5.50 -4.75 x008<br>OS: +5.75 -5.00 x172	Central
**AS1**	36, F	Strab	OD: 20/20<br>OS: 20/20 -1	Far: 4 RET<br>Near: 4 RET	OD: -0.50 DS<br>OS: -0.25 DS	Central
**AS2**	30, F	Strab	OD: 20/20 -1<br>OS: 20/20 -1	Far: 2 LET<br>Near: 4 LET	OD: +3.25 -1.25 x170<br>OS: +3.50 -1.25 x010	Central
**AS3**	20, M	Strab	OD: 20/20 -2<br>OS: 20/20 -1	Far: trace XP<br>Near: 6 XP	OD: +0.50 -0.50 x180<br>OS: plano -1.00 x180	Central
**AS4**	54, M	Strab	OD: 20/25 -1<br>OS: 20/20 -1	Far: 14 RET 5 HoT<br>Near: 6 LET 10HyT	OD: -1.00 -0.75 x160<br>OS: -1.00 -1.00 x020	Central
**ASW1**	22, F	Normal	OD: 20/20 +1<br>OS: 20/16 -2	Far: trace XP<br>Near: 2 XP	OD: -4.25 -1.00 x180<br>OS: -4.00 -0.75 x160	Central
**N1**	25, F	Normal	OD: 20/16 -1<br>OS: 20/16 -1	Far: ortho<br>Near: ortho	OD: -3.75 -0.75 x174<br>OS: -4.25 -0.50 x003	Central
**N2**	26, F	Normal	OD: 20/20<br>OS: 20/20	Far: ortho<br>Near: ortho	OD: -6.25 -0.25 x085<br>OS: -6.25 -0.25 x085	Central
**N3**	28, M	Normal	OD: 20/20 -2<br>OS: 20/20	Far: ortho<br>Near: ortho	OD: plano<br>OS: plano	Central
**N4**	24, F	Normal	OD: 20/25 +1<br>OS: 20/25 +2	Far: ortho<br>Near: 2XP	OD: plano<br>OS: plano	Central
**N5**	28, F	Normal	OD: 20/20 +2<br>OS: 20/20 +2	Far: ortho<br>Near: ortho	OD: -0.50 -2.25 x107<br>OS: -0.50 -2.25 x078	Central
**N6**	23, F	Normal	OD: 20/16 -2<br>OS: 20/16 -1	Far: trace XP<br>Near: 3 XP	OD: -3.25<br>OS: -3	Central
**N7**	23, F	Normal	OD: 20/20 +2<br>OS: 20/20	Far: trace XP<br>Near: 3 XP	OD: +0.25 -1.50 x177<br>OS: +0.25 -1.25 x176	Central
**N8**	24, F	Normal	OD: 20/20 -1<br>OS: 20/20	Far: 2 XP<br>Near: 6 XP	OD: -5.25<br>OS: -7.5	Central
**N9**	23, F	Normal	OD: 20/16 -1<br>OS: 20/16	Far: ortho<br>Near: 4 EP	OD: -1.00 DS<br>OS: -1.00 DS	Central
**N10**	24, F	Normal	OD: 20/16<br>OS: 20/12.5	Far: ortho<br>Near: ortho	OD: -1.75 DS<br>OS: -1.75 DS	Central

AA = Participants with anisometropia (n = 4); AMS = Micro-strabismic participant (n = 1); AS = Participants with strabismus (n = 4); ASW = Stereo-weak participant with otherwise normal binocular vision (n = 1); N = Stereo-normal participants (n = 10); * = participants with amblyopia.

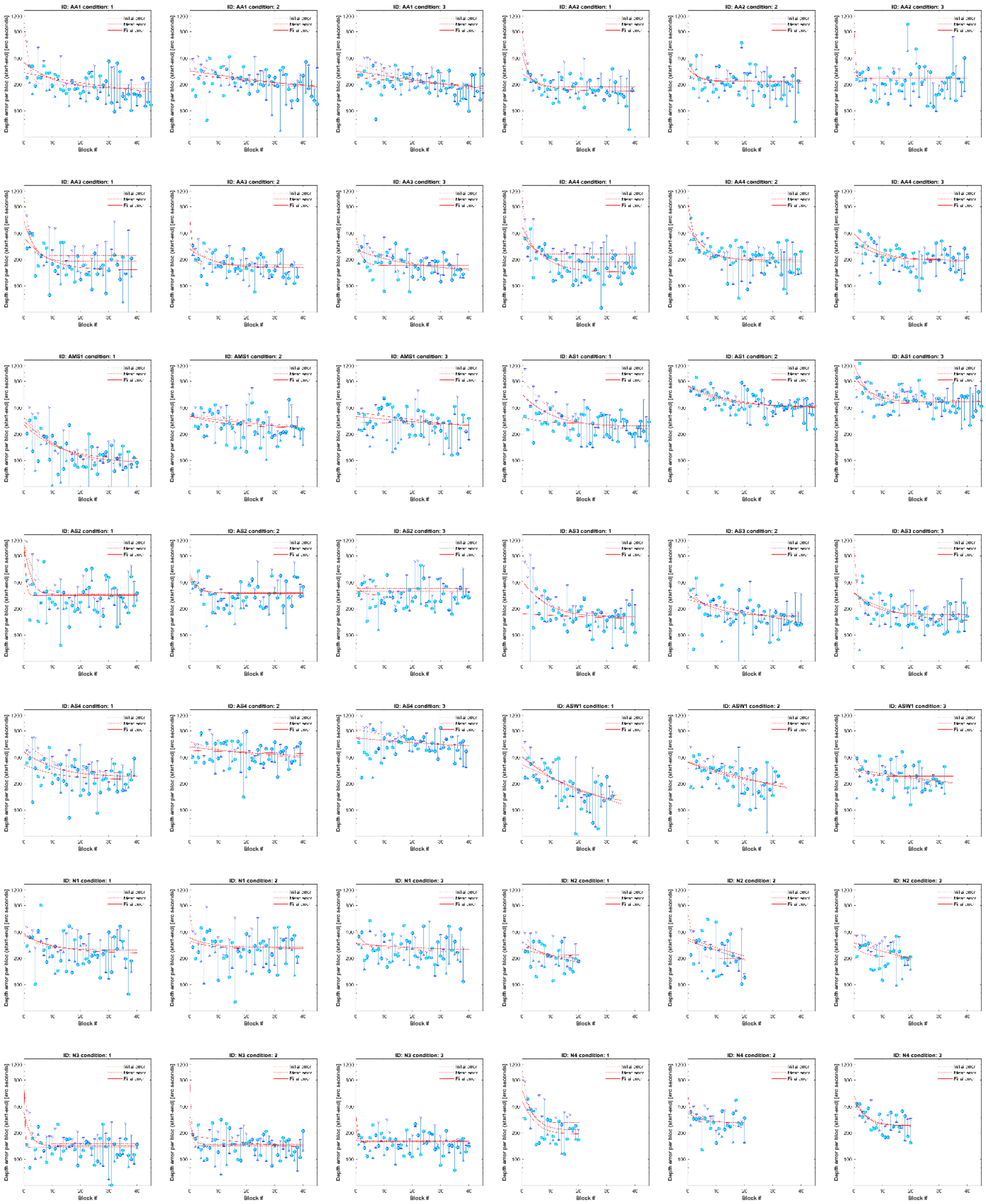
ID: AA1 condition: 1
ID: AA1 condition: 2
ID: AA1 condition: 3
ID: AA2 condition: 1
ID: AA2 condition: 2
ID: AA2 condition: 3
ID: AA3 condition: 1
ID: AA3 condition: 2
ID: AA3 condition: 3
ID: AA4 condition: 1
ID: AA4 condition: 2
ID: AA4 condition: 3
ID: AMS1 condition: 1
ID: AMS1 condition: 2
ID: AMS1 condition: 3
ID: AS1 condition: 1
ID: AS1 condition: 2
ID: AS1 condition: 3
ID: AS2 condition: 1
ID: AS2 condition: 2
ID: AS2 condition: 3
ID: AS3 condition: 1
ID: AS3 condition: 2
ID: AS3 condition: 3
ID: AS4 condition: 1
ID: AS4 condition: 2
ID: AS4 condition: 3
ID: ASW1 condition: 1
ID: ASW1 condition: 2
ID: ASW1 condition: 3
ID: N1 condition: 1
ID: N1 condition: 2
ID: N1 condition: 3
ID: N2 condition: 1
ID: N2 condition: 2
ID: N2 condition: 3
ID: N3 condition: 1
ID: N3 condition: 2
ID: N3 condition: 3
ID: N4 condition: 1
ID: N4 condition: 2
ID: N4 condition: 3
Depth error per bloc (start-end) [arc seconds]
Block #

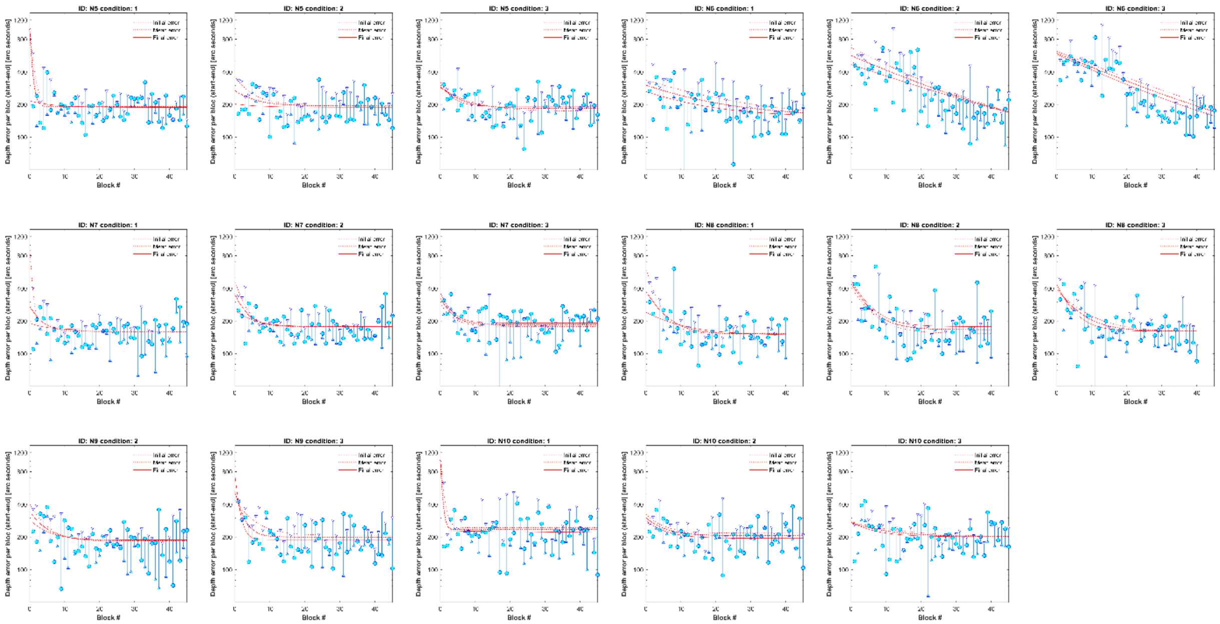

Figure S1. Across-block learning for each participant and condition. Each block is represented as a vertical line, with a triangle on one end indicating the depth error at the beginning of the block and a circle indicating the depth error at the end of the block. The fits represent an exponential function to the initial error at each block (dotted line), mean error (hashed line), and final error (continuous line).

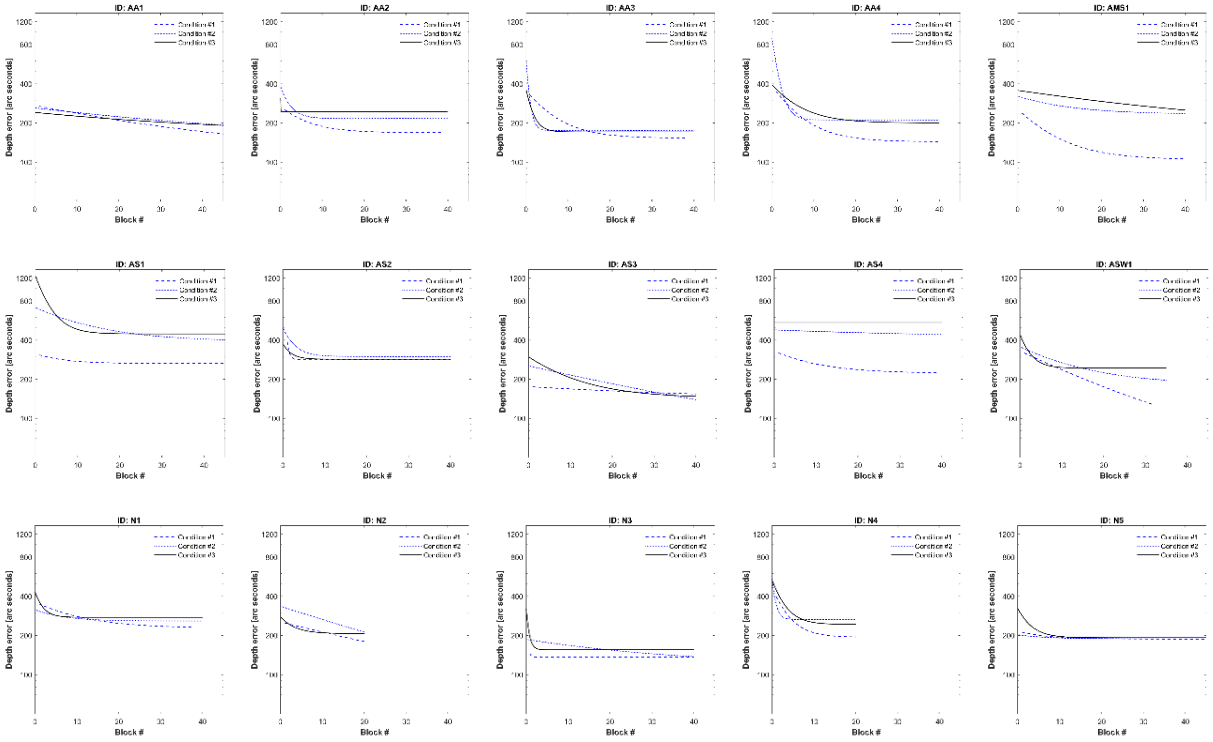

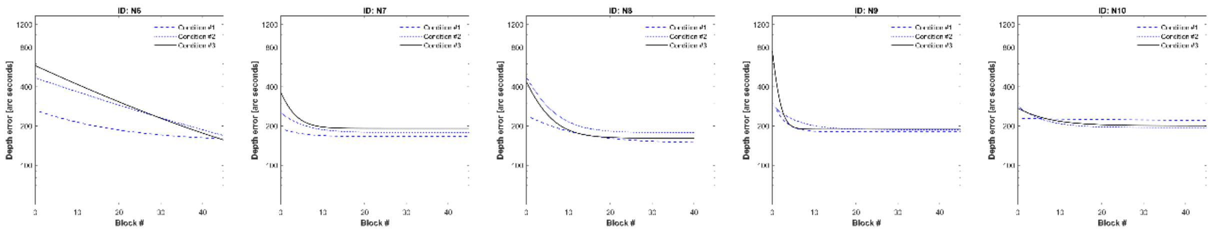

Figure S2. Across-block learning for all participants. Each graph shows the exponential fit of the end-block depth error in the three conditions: Condition 1, blue dashed line; Condition 2, blue dotted line; Condition 3, dark continuous line.

Table S2

DartBoard in-game accuracy of a normal sighted participant in two scenarios: binocular vision (BV) and monocular vision (MV) by means of a physical patch. Time of game play 15 minutes in both cases. Condition (Level 1, 2 and 3); total number of trials; percentage of successful hits towards de board; median accuracy and interquartile range (seconds of arc); dichoptic task errors (per 100 trials)

	Trials		Hits (per 100 trials)		Accuracy (arc secs)		Dichoptic errors	
Condition	BV	MV	BV	MV	BV	MV	BV	MV
1	23	14	100	78.6	201 [106 – 333]	583 [308 – 753]	0	14
2	22	19	100	84.2	251 [157 – 396]	566 [402 – 915]	0	26
3	24	19	100	63.2	165 [56 – 331]	363 [230 – 860]	42	53

Table S3

Halloween in-game accuracy of a normal sighted participant in two scenarios: binocular vision (BV) and monocular vision by means of a physical patch (MV). Time of game play 15 minutes in both cases. Condition (level 1, 2 and 3); total number of trials; successful trials (per 100 trials); dichoptic task errors (per 100 trials)

		Trials			Successful trials (per 100 trials)			Dichoptic errors (per 100 trials)		
		Cond 1	Cond 2	Cond 3	Cond 1	Cond 2	Cond 3	Cond 1	Cond 2	Cond 3
Overall	BV	146	141	133	92	89	86	2	1	3
	MV	133	136	118	77	61	65	10	10	12
1000”	BV	18	18	18	100	100	100	0	0	0
	MV	18	22	26	100	82	69	0	0	0
650”	BV	18	20	19	100	90	95	0	5	5
	MV	19	114	44	95	57	70	5	12	18
420”	BV	19	18	22	95	100	77	5	0	14
	MV	21		48	86		58	10		13
270”	BV	24	27	25	100	89	96	0	0	0

	MV	33			73			9		
180”	BV	25	27	26	96	85	92	4	0	0
	MV	42			60			17		
120”	BV	31	31	23	77	77	61	3	0	0
	MV									
80”	BV	11			82			0		
	MV									

Table S4

d’ PPR for each participant, cue scaffolding level, and stereoacuity demand.

SID	Condition 1				Condition 2				Condition 3			
	1000”	800”	600”	400”	1000”	800”	600”	400”	1000”	800”	600”	400”
AA1	1	1.28	1.07	0.93	0.84	1.1	1.29	1.29	1	1.01	1.11	0.93
AA2	1.15	1.11	1.18	1.45	1.11	1.11	1.02	1.5	0.8	1.25	1.63	2.05
AA3	0.99	1.10	1.17	1.29	1	1.06	1.18	0.91	0.99	1.12	1.24	1.22
AA4	0.99	2.37	1.55	1.49	0.99	2.06	1.83	1.3	1	1.53	1.63	0.77
AMS1	1	1.48	0.97	1.08	1	1.18	1.22	1.1	1	1.37	1.36	1.16
AS1	1	1.71	1.42	1.77	1.59	1.4	1.5	1.7	1.6	1.3	2	0.76
AS2	0.9	3.65	2.63	1.91	1	3.04	2.43	1.1	1.08	3.07	2.92	1.18
AS3	1	1.04	1.03	1.34	0.76	0.9	0.96	1.19	0.76	0.88	0.89	1
AS4	1	1.45	1.45	-	2.28	1.79	-	-	1.35	1.53	0.38	-
ASW1	1.01	1.15	1.14	1.18	1.2	1.2	0.97	1.1	1.12	1.1	1.16	0.88
N1	0.9	1.11	1.12	1.1	1.01	0.87	1.15	1.2	0.94	0.74	1.12	1.22
N2	1.01	1.4	1.4	1.05	1	1.19	1.38	1.46	1.06	1.27	1.31	0.95
N3	-	1.03	0.98	1.09	-	0.66	1.06	0.85	-	0.76	0.91	1.17
N4	1.12	1.4	1.36	1.35	1.25	1.45	1.28	2.2	1.07	1.2	1.5	0.54
N5	1	0.98	1.24	1.13	1	0.92	1.2	0.97	1	1.05	1.23	1.02
N6	1	1.05	1.04	0.88	1	1.02	1.22	1.37	1	1.17	1.27	1.31
N7	0.9	0.85	1.17	1	0.74	0.75	0.87	1.08	0.63	0.7	0.84	0.82
N8	1.06	1.33	1.43	0.83	1.22	1.14	1.13	1.27	1.22	0.95	1.26	1.64
N9	-	0.92	1.75	129	-	0.99	1.01	1	-	0.73	1.05	1.19
N10	1	0.93	1.11	1.53	1.01	0.85	1.07	1.06	1.01	0.92	1.07	1.02

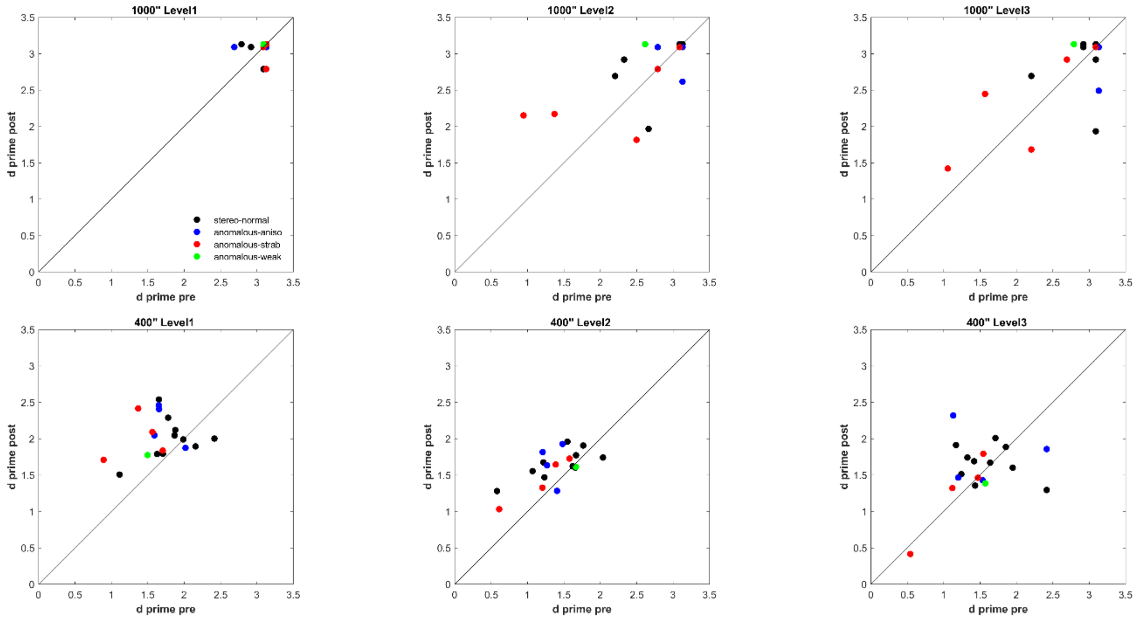

Figure S3. Halloween game pre post d' values for stereo demand of 1000" and 400" at each condition (level 1, 2 and 3). Symbols represent a different participant and group: anisometropia (blue), strabismus (red), stereo-weak (green), and normal binocular (grey). Points above the unity line indicate an improvement in accuracy in the first three hours compared to the last three hours of game play.
